# Osteopontin Exacerbates High-Fat Diet-Induced Metabolic Disorders in a Microbiome-Dependent Manner

**DOI:** 10.1128/mbio.02531-22

**Published:** 2022-10-27

**Authors:** Jianing Chen, Ping Zeng, Lan Gong, Xujun Zhang, Zongxin Ling, Kefan Bi, Fan Shi, Kaihang Wang, Qiong Zhang, Jingjing Jiang, Yanhui Zhang, Toshimitsu Uede, Emad M. El-Omar, Hongyan Diao

**Affiliations:** a State Key Laboratory for Diagnosis and Treatment of Infectious Diseases, National Clinical Research Center for Infectious Diseases, Collaborative Innovation Center for Diagnosis and Treatment of Infectious Diseases, The First Affiliated Hospital, College of Medicine, Zhejiang University, Hangzhou, China; b Jinan Microecological Biomedicine Shandong Laboratory, Jinan, China; c Microbiome Research Centre, St George and Sutherland Clinical School, University of New South Wales, Sydney, Australia; d Division of Molecular Immunology, Institute for Genetic Medicine, Hokkaido University, Sapporo, Japan; University of Chicago

**Keywords:** osteopontin, high-fat diet, metabolic disorder, gut microbiome, adhesion

## Abstract

The gut microbiome is involved in metabolic disorders. Osteopontin (OPN), as a key cytokine, contributes to various inflammation-related diseases. The underlying role of OPN in the microbiome remains poorly understood. Here, we investigated whether OPN could modulate metabolic disorders by affecting gut microbiota. In our present study, we found that the expression of OPN was elevated in individuals with obesity compared to that observed in healthy controls. There was a positive correlation between plasma OPN levels and body mass index (BMI) in humans. Moreover, OPN significantly exacerbated lipid accumulation and metabolic disorders in high-fat diet (HFD)-fed mice. Importantly, OPN significantly aggravated HFD-induced gut dysbiosis with a key signature profile. Fecal microbiota transplantation also supported the role of OPN in HFD-induced metabolic disorders in a microbiota-dependent manner. Moreover, the microbiome shift of OPN-deficient mice would be compensated to resemble those of wild-type mice by feeding with either OPN-containing milk or recombinant OPN protein *in vivo*. Furthermore, metagenomic analysis showed that OPN induced a higher abundance of *Dorea* and a lower abundance of *Lactobacillus*, which were positively and negatively correlated with body weight, respectively. Indeed, the abundance of *Dorea* was significantly decreased after *Lactobacillus* administration, suggesting that OPN may regulate the intestinal abundance of *Dorea* by reducing the colonization of *Lactobacillus*. We further confirmed that OPN decreased the adhesion of *Lactobacillus* to intestinal epithelial cells through the Notch signaling pathway. This study suggested that OPN could exacerbate HFD-induced metabolic dysfunctions through the OPN-induced alteration of the gut microbiome. Therefore, OPN could be a potential therapeutic target for metabolic syndrome.

## INTRODUCTION

Due to drastic changes in human lifestyle over the past century, metabolic disorders are becoming a global epidemic risk for public health. Metabolic disorders include the interrelated metabolic constellation of dyslipidemia, insulin resistance, and hepatic steatosis. More recent studies showed that the intestinal microbiome plays a crucial role in metabolic disorders (1, 2). The gut microbiome is also sensitive to dietary factors as well as other host factors. For example, obesity and insulin resistance were reported in germfree (GF) mice treated with Enterobacter cloacae on a high-fat diet (HFD) (3). Probiotics such as Lactobacillus could inhibit chylomicron secretion from enterocytes and promote lipid storage (4). Furthermore, fecal microbiota transplantation (FMT) from HFD-fed mice to GF mice led to a significant gain of weight and severe metabolic disorders in the recipient mice (5). In addition, the alteration of the microbiome has influence on many host pathways, such as those for energy regulation and lipid metabolism (6, 7), suggesting gut microbiota as a potential target for dyslipidemia therapy. However, until now, these intervention strategies, including FMT, are not always effective, and this might be due to the interference of the host factors.

Osteopontin (OPN), as a key cytokine, is present in the blood and milk of mammals. OPN is involved in chronic inflammatory diseases, including hepatitis, cancer, and other immune diseases (8–12). For instance, OPN-deficient mice displayed less severe hepatic steatosis (13). A reduced absorption of cholesterol was also observed in OPN-deficient mice, and this prevented gallstone formation (14). Furthermore, the level of OPN is regarded to be increased in obesity-associated diseases (15, 16). These studies indicated that OPN may influence the absorption and metabolism of lipids. This link, combined with the previously reported gut-lipid-metabolic disorder axis, leads to our hypothesis that OPN might affect metabolic disorders by altering the gut microbiome and lipid metabolism.

In this study, we first found that OPN levels were significantly higher in subjects with obesity and that a positive correlation existed between OPN levels and body mass index (BMI) in humans. Next, we confirmed this OPN-lipid metabolism link in a mouse model and investigated whether and how OPN modulated lipid metabolism. We demonstrated that there was a resistance to HFD-induced dyslipidemia in the OPN-deficient mice, in which OPN regulated the gut microbiome to influence lipid metabolism. Interestingly, FMT experiments further confirmed the effect of OPN on the gut microbiome in HFD-induced dyslipidemia. Cross-fostering experiments showed that feeding OPN-deficient infant mice with OPN-containing milk resulted similar microbiome communities to those of wild-type mice. Finally, we suggested a possible mechanism of OPN modulating microbiota, in which OPN decreased the adhesion of the typical probiotic bacteria *Lactobacillus* to intestinal epithelial cells by inhibiting the production of adhesion molecules. This study showed that OPN could contribute to metabolic disorders through the OPN-induced alteration of the gut microbiome and lipid metabolism.

## RESULTS

### Requirement of OPN in high-fat diet-induced lipid accumulation.

**FIG 1:**
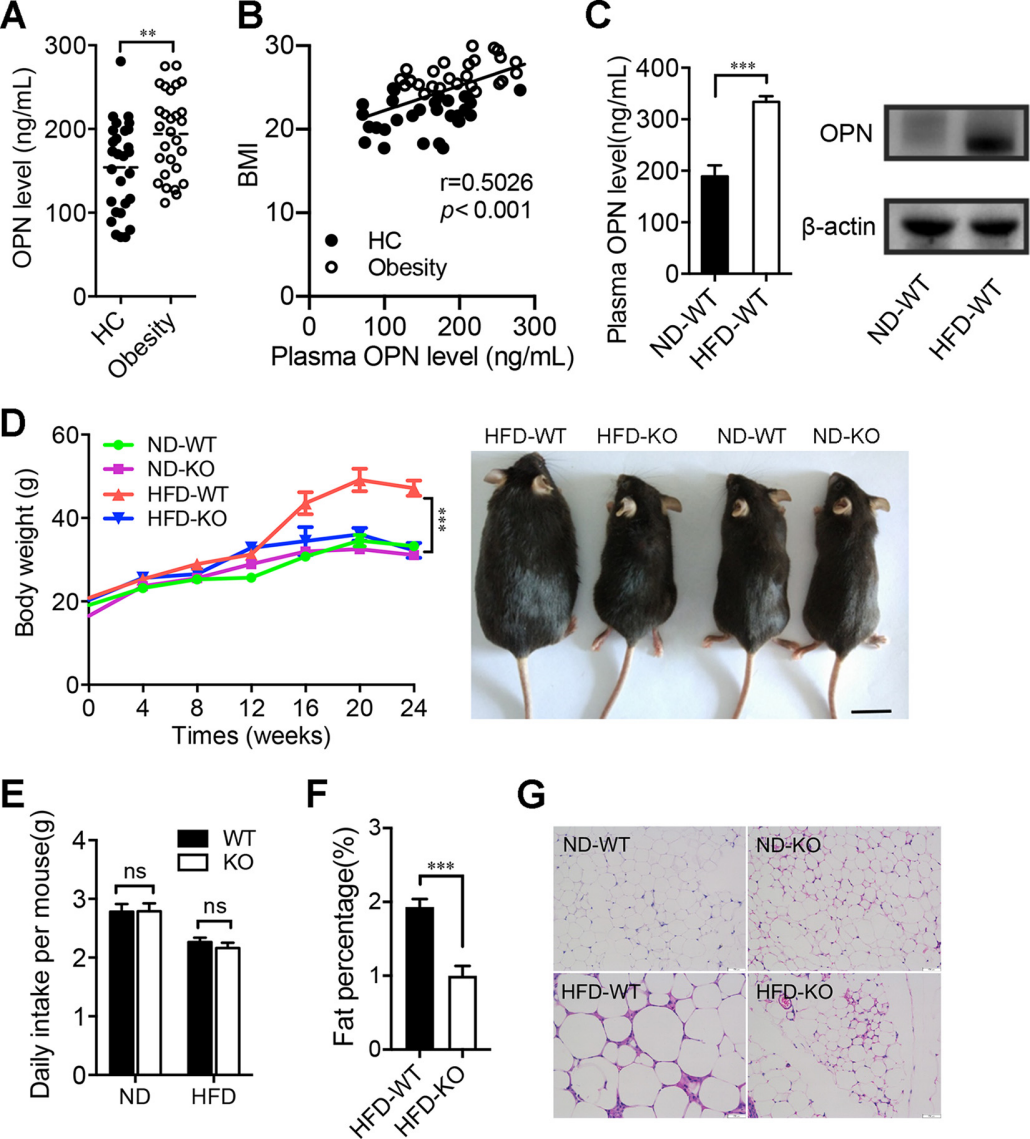
Osteopontin (OPN) deficiency resisted the high-fat diet (HFD)-induced lipid accumulation. The plasma samples of individuals with obesity (Obesity, *n* = 28) and nonobese healthy controls (HC, *n* = 27) were collected. (A) Plasma OPN levels and (B) the correlation of BMI and plasma OPN levels were assessed. Then, wild-type (WT) and OPN knockout (KO) mice were fed with a normal diet (ND) or a high-fat diet for 24 weeks. (C) The plasma and intestinal OPN levels in WT mice were measured after 24-weeks of ND-feeding or HFD-feeding (ND-WT, HFD-WT). (D) Dynamic observation of body weight at week 0 to 24 and the representative image of mice at week 24 in ND-fed or HFD-fed WT and KO mice (ND-WT, ND-KO, HFD-WT, HFD-KO). (E) Comparison of daily diet intake per mouse among the four groups of ND-WT, ND-KO, HFD-WT and HFD-KO mice. (F) Fat percentage (%) in HFD-WT and HFD-KO mice. (G) Representative histological images of hematoxylin-eosin (HE) staining on adipose tissues in mice (ND-WT, ND-KO, HFD-WT, HFD-KO). Data are given as the means ± the standard errors of the mean (SEMs). A Student’s *t* test (panels A, C, and F), the Spearman’s rank correlation (panel B), and a two-way analysis of variance (ANOVA) (panels D and E) were used to analyze the data with *n* = 8 to 12 per group, **, *P* < 0.01; ***, *P* < 0.001.

Since it has been shown that OPN is involved in lipid metabolic-associated fatty liver disease (13, 17), we first compared the human plasma levels of OPN between individuals with obesity and healthy controls (HC). Consistent with the previous study (18), the OPN levels were significantly higher in subjects with a high body mass index (BMI) than those measured in controls with a normal BMI (HC) (Fig. 1A). Moreover, there was a positive correlation between human OPN levels and BMI (Fig. 1B). To determine whether OPN also contributed to lipid accumulation in a mouse model, we measured the OPN levels in different tissues of wild-type (WT) mice fed with a high-fat diet (HFD) or a normal diet (ND). The levels of plasma and intestinal OPN were both significantly increased in the HFD-WT mice compared to the ND-WT mice (Fig. 1C). Next, we found that HFD significantly increased body weight in WT mice from 12 to 24 weeks but failed to do so in OPN-KO mice (Fig. 1D; Fig. S1A). No significant difference in food intake was observed between the two groups (HFD-WT versus HFD-KO), suggesting that food consumption was not the explanation for the effects of OPN on weight gain and lipid accumulation (Fig. 1E). Magnetic resonance imaging (MRI) revealed an increased fat mass in the abdomen of the HFD-WT mice compared to that of HFD-KO mice (Fig. 1F; Fig. S1B). Furthermore, an assessment of abdominal adipose tissue indicated that HFD induced more lipid accumulation and the enlargement of adipocytes in HFD-WT mice, while no significant changes were observed in the OPN-KO mice fed with HFD (Fig. 1G; Fig. S1C and D). Taken together, these results indicate the involvement of OPN in HFD-induced lipid accumulation.

10.1128/mbio.02531-22.4 FIG S1 More accumulated lipid in WT mice fed with HFD. (A) The level of body weight was detected in the WT and KO mice at week 24 with ND or HFD administration. (B) T1-weighted MRI measurement in HFD-WT and HFD-KO mice. (C and D) The weight and the size of abdominal adipose tissue were determined in WT and KO mice fed with ND or HFD. (E) Representative histological images of HE staining of liver in four groups. Original magnification, scale bars, 50 μm. Data are given as mean ± SEM. A two-way ANOVA (panels A, C) was used to analyze the data with *n* = 8 to 12 per group. ***, *P* < 0.001. Download FIG S1, TIF file, 2.6 MB. Copyright © 2022 Chen et al. 2022 Chen et al. https://creativecommons.org/licenses/by/4.0/ This content is distributed under the terms of the Creative Commons Attribution 4.0 International license.

### OPN deficiency exhibited metabolic protective effects in HFD-fed mice.

**FIG 2:**
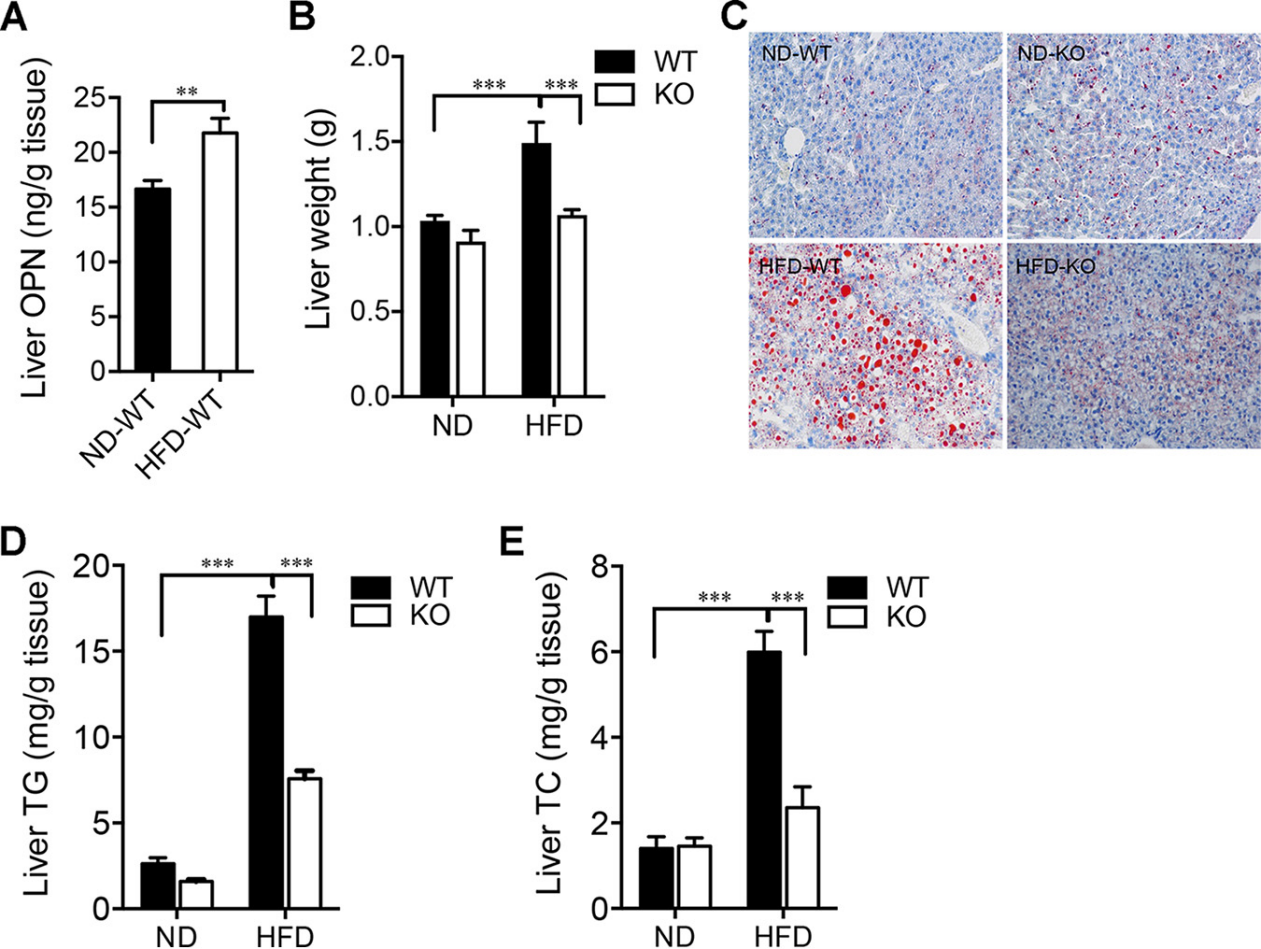
OPN aggravated liver lipid metabolic disorders in HFD-fed mice. Liver was obtained from WT or KO mice at week 24 of feeding with ND or HFD. (A) Hepatic OPN levels in WT mice were measured after week 24 of ND-feeding or HFD-feeding (ND-WT, HFD-WT). (B) Liver weights were compared between the ND or HFD-fed WT and KO mice. (C) Representative histological images of lipid content assessed with Oil Red O staining. Original magnification, scale bars, 50 μm. Comparison of (D) liver triglyceride (TG) and (E) cholesterol (TC) levels in the ND or HFD-fed WT and KO mice. A Student’s *t* test (panel A) and a two-way ANOVA (panels B, D, and E) were used to analyze the data. Data are given as the mean ± SEM. *n* = 6 per group, **, *P* < 0.01; ***, *P* < 0.001.

In the liver, HFD induced an elevated level of OPN (Fig. 2A). In addition to interference with lipid homeostasis, there were significantly increased liver weight and lipid accumulation in HFD-WT mice compared to those in the other three groups: HFD-KO, ND-WT, and ND-KO (Fig. 2B and C; Fig. S1E). The hepatic levels of related indicators of glycolipid metabolism, such as total cholesterol (TC) and triglycerides (TG), were also markedly higher in the HFD-WT mice compared to the other three groups (Fig. 2D and E). Furthermore, we measured the plasma levels of TC, TG, high-density lipoprotein (HDL), and low-density lipoprotein (LDL). Indeed, these lipid metabolic parameters were significantly increased in the HFD-WT mice compared to those observed in the HFD-KO mice (Table 1). Compared with the HFD-KO mice, there were significantly increased levels of blood glucose, insulin, and leptin in the HFD-WT mice with decreased glucose tolerance and impaired insulin sensitivity, while the OPN^−/−^ mice were resistant to the disorder of glucolipid metabolism induced by HFD (Table 1; Fig. S2A and B). These results collectively suggest a resistance to HFD-induced lipid accumulation and metabolic disorders in the OPN-deficient (KO) mice.

**TABLE 1:** Biochemical data of serum parameters in the four groups of mice^*a*^

Serum parameter	ND-WT	ND-KO	HFD-WT	HFD-KO	ND-WT vs HFD-WT	HFD-WT vs HFD-KO	HFD-KO vs ND-KO vs ND-WT
Triglycerides (mg/dL)	114.0 ± 10.16	91.2 ± 10.56	225.8 ± 18.47	84.0 ± 1.45	***	***	ns
Total cholesterol (mg/dL)	104.4 ± 8.10	87.5 ± 6.30	244.0 ± 20.65	103.8 ± 1.93	***	***	ns
HDL (mg/dL)	75.3 ± 7.21	62.7 ± 4.84	232.0 ± 24.73	83.3 ± 3.42	***	***	ns
LDL (mmol/L)	0.1 ± 0.01	0.2 ± 0.02	1.2 ± 0.07	0.3 ± 0.04	***	***	ns
Glucose (mmol/L)	6.6 ± 0.64	6.9 ± 0.54	10.3 ± 1.12	7.8 ± 0.54	*	ns	ns
Insulin (ng/mL)	0.7 ± 0.18	0.7 ± 0.13	1.7 ± 0.02	0.4 ± 0.07	***	***	ns
Leptin (ng/mL)	13.2 ± 4.26	4.8 ± 0.73	133.3 ± 5.33	56.2 ± 14.98	***	***	ns

a Student’s *t* test, *, *P* < 0.05; ***, *P* < 0.001; ns, non significant.

10.1128/mbio.02531-22.5 FIG S2 OPN aggravated lipid metabolic disorders in HFD-WT mice. Liver was obtained after 24 weeks of the administration of the ND or HFD diet. Effect of OPN deficiency on the percentage (area under the curve, AUC) of initial blood glucose levels during (A) insulin tolerance test (ITT), (B) glucose tolerance test (GTT), (C and D) serum TG and HDL levels during an oral lipid tolerance test (OLTT). (E and F) Synthases (FAS, ACC, and HMG-CoAR) and lipidolytic enzymes (ACO, CAT, and AMPK) were both analyzed and compared in HFD-fed WT and KO mice. Data are given as mean ± SEM. Two-way ANOVAs were used in panels A–D, and two-tailed Student’s *t* tests were used in panels C–F. *n* = 4−6 per group. *, *P* < 0.05; ***, *P* < 0.001. Download FIG S2, TIF file, 0.4 MB. Copyright © 2022 Chen et al. 2022 Chen et al. https://creativecommons.org/licenses/by/4.0/ This content is distributed under the terms of the Creative Commons Attribution 4.0 International license.

### OPN facilitated HFD-induced intestinal metabolic dysfunction.

To address the role of OPN in the digestion of lipids, we performed an oral lipid tolerance test, and the results of fat absorption showed no difference between the two groups of HFD-fed mice (WT and OPN-KO) (Fig. S2C and D). In addition, we also analyzed and compared the levels of lipid synthetase and lyase in the livers of the two groups of HFD-fed mice. There was no difference in the levels of these lipid metabolic enzymes between these two groups (Fig. S2E and F).

To investigate whether OPN deficiency protects from dyslipidemia by mediating the metabolism of lipids in mice, a transcriptome analysis was conducted using the intestine tissue collected from two groups of HFD-fed mice (WT and OPN-KO). By performing a principal components analysis (PCA), the samples were separated into two distinct subgroups based on the presence or absence of OPN (HFD-WT versus HFD-KO) (Fig. S3A). A Venn diagram analysis showed that the fragments per kilobase of transcript per million mapped reads (FPKM) were greater than 1 in the HFD-WT group and the HFD-KO group. We found that the total numbers of expressed genes in the HFD-WT group and the HFD-KO group were 11,353 and 11,573, respectively, of which 10,993 were shared by both groups (Fig. S3B). There were 764 differentially expressed genes, including 293 genes upregulated and 471 genes downregulated, in the HFD-WT mice compared to the HFD-KO mice (Fig. S3C). A heat map showed all of the differentially expressed genes in both groups (Fig. S3D). Next, using a Gene Ontology (GO) enrichment analysis, we found that the majority of the 293 upregulated genes in the HFD-WT mice were associated with lipid biosynthetic, lipid catabolic, or fatty acid metabolism processes (Fig. S3E). These upregulated genes were also involved in Kyoto Encyclopedia of Genes and Genomes (KEGG) pathways, including those for retinol metabolism, linoleic acid metabolism, arachidonic acid metabolism, and insulin resistance, as well as the peroxisome proliferator-activated receptor (PPAR) signaling pathway (Fig. S3F).

10.1128/mbio.02531-22.6 FIG S3 Transcriptome analysis in intestine tissue collected from two groups of HFD-fed mice. (A) PCA, (B) Venn diagram, (C) volcano map, (D) cluster analysis of differentially expressed genes, (E) KEGG signaling pathway analysis, and (F) GO biological process cluster analysis in the intestine tissue of HFD-KO and HFD-WT mice. Download FIG S3, TIF file, 0.4 MB. Copyright © 2022 Chen et al. 2022 Chen et al. https://creativecommons.org/licenses/by/4.0/ This content is distributed under the terms of the Creative Commons Attribution 4.0 International license.

Then, we determined the fecal contents of long-chain fatty acids (LCFAs) and short-chain fatty acids (SCFAs) in the WT and OPN-KO mice. The levels of total fecal LCFAs and many individual ones were significantly higher in the HFD-KO mice than those observed in the HFD-WT mice (Fig. S4A–C), whereas there was no significant difference in the levels of total and individual fecal LCFAs between the two groups of ND-fed mice (ND-WT versus ND-KO) (Fig. S4D and E). We also found that the level of total fecal SCFAs in the HFD-fed mice was significantly lower than that observed in the ND-fed mice, with a nonsignificant reduction in the OPN-KO mice compared to the ND-fed or the HFD-fed WT mice (Fig. S4F). Taken together, the transcriptome data show that OPN actively regulates the metabolism of intestinal lipids and fatty acids in the HFD-fed mice.

10.1128/mbio.02531-22.7 FIG S4 The levels of individual long-chain fatty acids. (A) The levels of the total long-chain fatty acids (LCFAs) were measured in the feces of the HFD-fed WT and KO mice. (B) Individual level of fecal LCFA was determined in the HFD-WT and HFD-KO mice. (C) The heat-map of fecal LCFAs between HFD-WT and HFD-KO mice. (D) The total and (E) the individual long-chain fatty acids were determined in the ND-fed WT and KO mice. (F) The total short-chain fatty acids were determined in ND-fed or HFD-fed WT and OPN-KO mice. Data are given as mean ± SEM. A two-tailed Student’s *t* test was used to analyze the data with *n* = 8 to 12 per group. *, *P* < 0.05; **, *P* < 0.01; ***, *P* < 0.001. Download FIG S4, TIF file, 1.0 MB. Copyright © 2022 Chen et al. 2022 Chen et al. https://creativecommons.org/licenses/by/4.0/ This content is distributed under the terms of the Creative Commons Attribution 4.0 International license.

### Role of OPN in HFD-induced gut microbiota dysbiosis.

**FIG 3:**
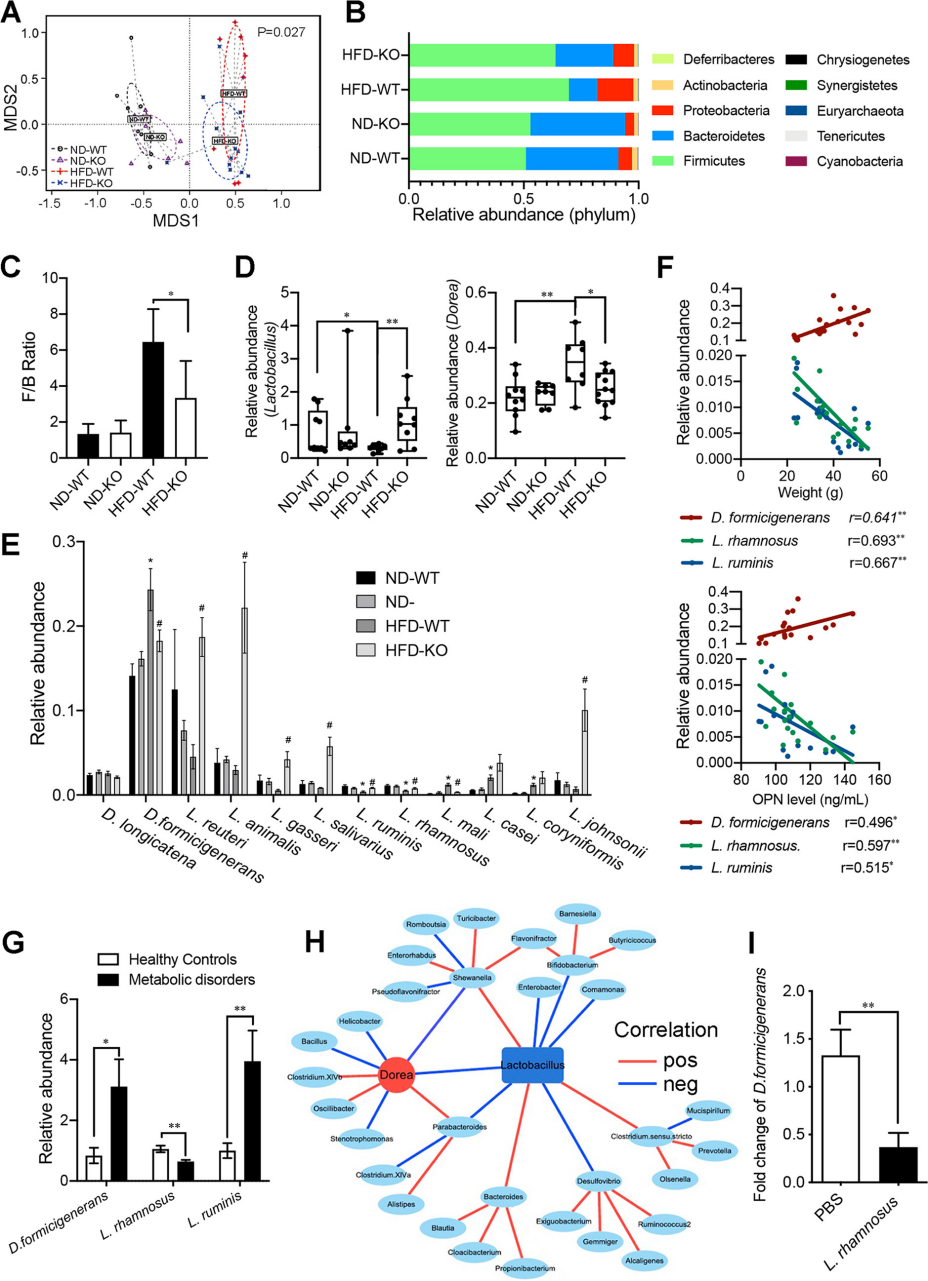
OPN significantly influenced the intestinal microbiota in HFD-fed mice. WT and OPN-KO mice were fed with ND or HFD for 24 weeks. (A) Microbial β-diversity among the four groups (ND-WT, ND-KO, HFD-WT, HFD-KO) was distinguished using Nonmetric Multidimensional scaling (NMDS) analysis. (B) Bacterial taxonomic profiling at the phylum level of luminal bacteria and (C) Firmicutes-to-Bacteroidetes (F/B) ratio in the indicated four groups. (D) The relative abundance of the bacterical genera *Lactobacillus* and *Dorea* were selected for comparison in the relative abundance among the four groups. (E) Species including D. formicigenerans, D. longicatena, L. reuteri, L. animalis, L. gasseri, L. salivarius, L. ruminis, L. rhamnosus, L. mali, L. casei, L. coryniformis, and L. johnsonii were selected for comparison in the relative abundance among the four groups. (F) The correlation was analyzed between the relative abundance of the three selected species *D. formicigenerans*, *L. ruminis*, and L. rhamnosus, and the levels of OPN and body weight. (G) In humans, the relative abundances of *D. formicigenerans*, *L. ruminis*, and L. rhamnosus were compared between the microbiota of individuals with metabolic disorders and the microbiota of healthy controls. (H) The correlation network of *Lactobacillus* and other gut bacterial genera (red, positive; blue, negative). (I) The fecal relative abundance of *D. formicigenerans* was detected at day 7, following the intragastric administration of L. rhamnosus (1 × 10^9^). Data are given as mean ± SEM. A Wilcoxon test (panels C–E), the Spearman's rank correlation (panel F), the Pearson correlation (panel H), and a Student’s *t* test (panels G, I) were used to analyze the data with *n* = 8 to 12 per group. * or ^#^, *P* < 0.05; **, *P* < 0.01.

Then, we tested whether the OPN-mediated HFD-induced disordered metabolism of lipids and fatty acids was due to OPN-induced alterations in the gut microbiome. First, we noticed a higher expression of intestinal OPN in the HFD-WT mice than in the ND-WT mice (Fig. 1C), suggesting a possible link between intestinal OPN expression and gut microbiota. Next, a nonmetric multidimensional scaling (NMDS) analysis of intestinal metagenome data indicated that OPN deficiencies influence the composition of the gut microbiota when mice were fed with HFD, but this result did not hold true in the ND-fed mice (Fig. 3A). To assess the overall composition of the gut bacterial community in these four groups, we analyzed the degree of bacterial taxonomic similarity at the phylum level (Fig. 3B). Compared to the ND-WT mice, the HFD-WT mice displayed a significant decrease in the relative abundance of Bacteroidetes and a higher Firmicutes-to-Bacteroidetes (F/B) ratio, while OPN deficiency (KO) protected against this effect to a large extent (Fig. 3B and C). Among the top 25 genera, the relative abundance of the genus *Dorea* was significantly higher in the HFD-WT mice than was observed in the ND-WT and HFD-KO groups, while that of genus *Lactobacillus* was significantly lower in the HFD-WT mice (Fig. 3D; Fig. S5). We analyzed the relative abundance of two *Dorea* species, D. formicigenerans and D. longicatena, as well as the top 10 *Lactobacillus* species: L. reuteri, L. animalis, L. gasseri, L. salivarius, L. ruminis, L. rhamnosus, L. mali, L. casei, L. coryniformis, and L. johnsonii. Among those, the relative abundance of *D. formicigenerans* was higher and the relative abundance of *L. ruminis* and L. rhamnosus were lower in the HFD-WT mice than in the ND-WT and HFD-KO mice, while those of other species were not (Fig. 3E). Also, the relative abundance of *D. formicigenerans* was positively correlated with body weight and the serum OPN levels (Fig. 3F). In contrast, the relative abundance of *L. ruminis* and L. rhamnosus were negatively correlated with those two levels (Fig. 3F).

10.1128/mbio.02531-22.8 FIG S5 Bacterial taxonomic profiling at the genus level from four different groups. Student’s *t* test with *n* = 8 to 12 per group. * and ^#^, *P* < 0.05. Download FIG S5, TIF file, 0.1 MB. Copyright © 2022 Chen et al. 2022 Chen et al. https://creativecommons.org/licenses/by/4.0/ This content is distributed under the terms of the Creative Commons Attribution 4.0 International license.

To verify these dyslipidemia-specific gut bacterial signatures in humans, we analyzed the involvement of three tested species (*D. formicigenerans*, *L. ruminis* and L. rhamnosus) in metabolic disorders. In concordance with the results from the mouse model (Fig. 3F), the fecal relative abundance of *D. formicigenerans* was higher and that of L. rhamnosus was lower in individuals with metabolic disorders compared to those observed in healthy controls (Fig. 3G). However, the results of *L. ruminis* in human metabolic disorders differed from the results based on the data from the HFD-fed mice (Fig. 3G). Interestingly, Wang et al. found that L. rhamnosus attenuated weight gain and markedly improved glucose–insulin homeostasis and hepatic steatosis (19). Here, we found that a negative correlation existed between the relative abundances of the bacterial genera *Lactobacillus* and *Dorea* and that *Dorea* is further associated with many other gut bacteria involved in OPN-associated HFD-induced dyslipidemia (Fig. 3H). To provide direct evidence for the negative regulation of *Lactobacillus* on the abundance of *Dorea*, we then tested whether the administration of L. rhamnosus *in vivo* could induce a decreased expansion of *D. formicigenerans*. Indeed, the abundance of *D. formicigenerans* in the HFD-fed mice was significantly decreased at day 7 following the intragastric administration of L. rhamnosus (Fig. 3I). Taken together, these results indicated that OPN aggravated HFD-induced metabolic disorders and that this might be due to OPN-induced gut microbiota dysbiosis via the enrichment of *D. formicigenerans* and the reduction of L. rhamnosus.

### OPN-deficiency alleviated HFD-induced metabolic disorders in a microbiota-dependent manner.

**FIG 4:**
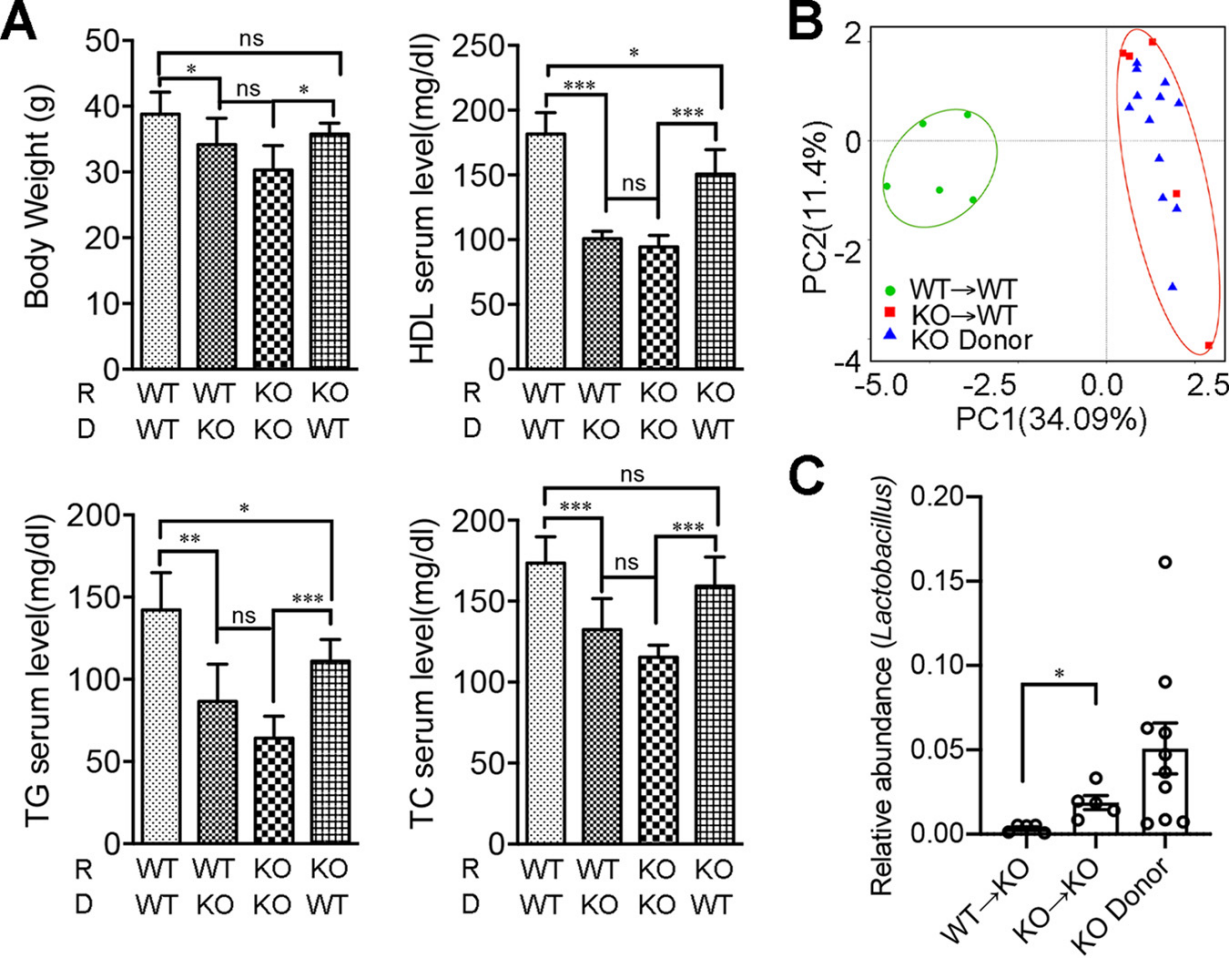
Fecal microbiota transplantation manipulated OPN-dependent lipid accumulation and metabolic disorders. Having been transplanted with the fecal microbiota from the donor HFD-WT or HFD-KO mice, the pseudosterile recipient WT or OPN-KO mice were fed with HFD for 16 weeks, continuously. (A) The levels of body weight, serum TC, TG, and LDL were analyzed in the four groups of recipient mice post FMT. (B) A principal components analysis (PCA) among the recipients of KO→WT, WT→WT, and the KO donor mice. (C) Relative abundance analysis of the genus *Lactobacillus* is shown in donor and recipient mice. Data are given as the mean ± SEM. A two-way ANOVA (panel A) and a Wilcoxon test (panel C) were used to analyze the data with *n* = 5-12 per group. *, *P* < 0.05; **, *P* < 0.01; ***, *P* < 0.001.

As gut dysbiosis is commonly involved in various metabolic disorders, we hypothesized that OPN might influence HFD-induced dyslipidemia by affecting microbiota. FMT experiments were performed to test this hypothesis, and in these experiments, the fecal microbiota of HFD-KO and HFD-WT mice were separately transplanted to HFD-WT or HFD-KO recipient mice following treatment with antibiotics. The body weight and serum lipid levels (TG, TC, HDL) were more similar in those recipients with the same donor mice after 16 weeks of continuous HFD feeding (Fig. 4A; Fig. S6A and B). The efficiency of the FMT was confirmed via a microbiome analysis of the donor and the recipient before and after the FMT. For example, the microbial β-diversity analysis conducted using a PCA showed a strong grouping by OPN-KO donor, which indicated that the microbial communities of the KO-to-WT FMT mice were closer to those of their KO donor mice than to those of their WT recipient mice (Fig. 4B). We further analyzed the relative abundance and the composition of the microbiota among those three groups: the WT recipient mice, the KO donor mice, and the KO-to-WT FMT mice. At the phylum and family levels, the composition of the microbiota in the FMT mice was more similar to that of the donor mice than to that of the recipient mice (Fig. S6C). We also found that the relative abundance of the bacterial genus *Lactobacillus* in the KO-to-KO FMT mice was significantly higher than that observed in the WT-to-KO FMT mice, which was similar to that observed in the KO donor mice (Fig. 4C). Taken together, this FMT experiment confirmed that OPN deficiency could inhibit HFD-induced dyslipidemia by modulating the gut microbiome.

10.1128/mbio.02531-22.9 FIG S6 Fecal microbiota transplantation manipulated OPN-dependent lipid accumulation and metabolic disorders. Transplanted with the fecal microbiota from the donor HFD-WT or HFD-KO mice, the pseudosterile recipient WT or OPN-KO mice were fed with HFD for 16 weeks, continuously. (A) Representative images of the HFD-fed recipient mice (WT→WT, WT→KO, KO→KO, KO→WT) after fecal microbiota transplantation (FMT). (B) The levels of body weight, serum TC, TG, and LDL were analyzed in four groups of recipient mice prior to the FMT. (C) Relative abundance analysis of dominant bacteria at the level of phylum or family is shown for donor and recipient mice. Data are given as mean ± SEM. A two-tailed Student’s *t* test was used to analyzed the data with *n* = 4 to 6 per group. *, *P* < 0.05; **, *P* < 0.01; ***, *P* < 0.001. Download FIG S6, TIF file, 0.9 MB. Copyright © 2022 Chen et al. 2022 Chen et al. https://creativecommons.org/licenses/by/4.0/ This content is distributed under the terms of the Creative Commons Attribution 4.0 International license.

### OPN modulated intestinal microbiome *in vitro* and *in vivo*.

**FIG 5:**
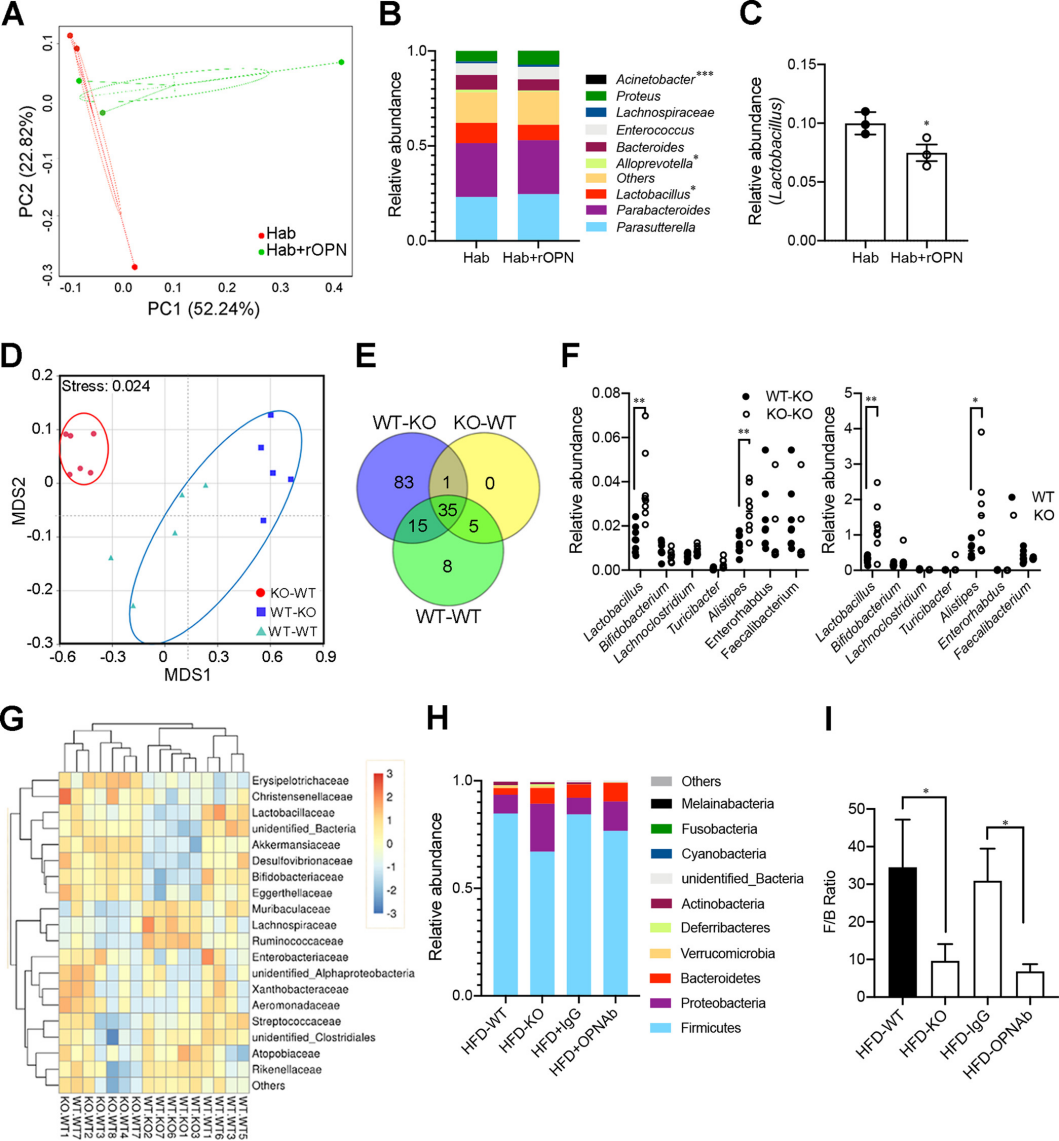
OPN directly altered the composition of the gut microbiota, including *Lactobacillus* attenuation. The isolated mixed OPN-KO fecal bacteria were cultured in hemolysin-containing anaerobic bottles (Hab) with or without recombinant OPN (rOPN) for 24 h before 16S rDNA amplicon sequencing. (A) A PCA plot of mixed microbial composition was assessed with or without rOPN in Hab broad-spectrum media (*n* = 3, three experiments). (B) Bacterial taxonomic profiling of the top 10 genera of intestinal bacteria isolated from OPN-KO mice with or without rOPN in Hab media. (C) Relative abundance analysis of the genus *Lactobacillus* was shown with or without rOPN in Hab media. The WT or OPN-KO lactating mice nursed the WT or KO infant mice for 3 weeks before the 16S rRNA sequencing of the intestinal luminal microbiota. (D) NMDS analysis showed the microbial β-diversity among the KO-WT (*n* = 6), WT-KO (*n* = 5), and WT-WT (*n* = 5) infant mice. (E) Venn diagram of the core microbiota communities (genus level) in the luminal of three groups of infant mice (WT-WT, WT-KO, and KO-WT). The core microbiota communities were present in at least 50% of the samples. (F) The difference in the relative abundance of the bacteria genera *Lactobacillus*, *Bifidobacterium*, *Lachnoclostridium*, *Turicibacter*, *Alistipes*, *Faecalibaculum*, and *Enterorhabdus* was compared between the KO-KO mice, the KO-WT infant mice, and the WT and KO lactating mice. (G) The heat map shows the abundance of the bacterial families in the feces of the WT-WT, WT-KO and KO-WT mice. (H) The HFD-WT mice were treated with anti-OPN antibodies or control IgG using an analysis of bacterial taxonomic profiling at the genus level and (I) the Firmicutes-to-Bacteroidetes (F/B) ratio in the HFD-WT, HFD-IgG, HFD-OPNAb, and HFD-KO groups. Data are given as the mean ± SEM. A Wilcoxon test (panels C, F) and a two-way ANOVA (panel I) were used to analyze the data with *n* = 3 to 8 per group. *, *P* < 0.05; **, *P* < 0.01; ***, *P* < 0.001.

In order to further illustrate the direct modulating effect of external OPN on the gut microbiome, we performed *in vitro* fecal bacterial coculture experiments with or without OPN treatment. The isolated fecal OPN-KO bacteria were cultured anaerobically in hemolysin-containing anaerobic bottles (Hab) with or without recombinant OPN (rOPN) for 24 h prior to 16S rDNA amplicon sequencing (Fig. S7A). A microbial β-diversity analysis indicated that OPN induced a significant microbiome shift and a decreased relative abundance of *Lactobacillus*, which was similar to those observed in the WT mice compared to those observed in the OPN-KO mice (Fig. 5A–C; Fig. 3D).

10.1128/mbio.02531-22.10 FIG S7 The OPN levels in both plasma and milk, as well as the in vivo and in vitro experiments of the effect OPN on the microbiome. (A) The isolated mixed OPN-KO fecal bacteria were cultured in hemolysin-containing anaerobic bottles (Hab) with or without recombinant OPN (rOPN) for 24 h before 16S rDNA amplicon sequencing. (B) Murine OPN was detected in both plasma (*n* = 6) and milk (*n* = 3). (C) The WT or OPN-KO lactating mice nursed the WT or KO infant mice for 3 weeks before the 16S rRNA sequencing of the intestinal luminal microbiota. Data are given as mean ± SEM, and a two-tailed Student’s *t* test was used to analyze the data. Download FIG S7, TIF file, 0.4 MB. Copyright © 2022 Chen et al. 2022 Chen et al. https://creativecommons.org/licenses/by/4.0/ This content is distributed under the terms of the Creative Commons Attribution 4.0 International license.

OPN is abundant in human breast milk (20). Actually, the OPN level in mouse milk was almost as high as that measured in the plasma (Fig. S7B). To further investigate whether feeding with external OPN regulates the intestinal microbiome *in vivo* in OPN-deficient mice, we analyzed the gut microbiota of WT or OPN-KO infant mice that were foster-nursed by WT and OPN-KO lactating mice, respectively (Fig. S7C). Interestingly, there were significant alterations in the microbial communities of the KO-WT mice (WT mice fed with KO milk) compared to those of the WT-WT and WT-KO mice (WT and KO mice fed with WT milk), based on whether the OPN protein was contained in the milk or not (Fig. 5D). A Venn diagram analysis showed that 15 genera were shared in the core microbiota communities between the WT-KO group and the WT-WT group, which were more than those (5 genera) shared between the KO-WT group and the WT-WT group (Fig. 5E). We found a similar trend of 7 shared genera, including *Lactobacillus*, in the intestinal microbiota between the KO-KO and KO-WT infant mice as well as the WT and KO adult mice (Fig. 5F). A heat map demonstrated that the abundances of the top 20 bacterial families in the WT-WT group were more similar to those in the WT-KO group than to those in the KO-WT group (Fig. 5G).

We further confirmed this OPN-mediated microbiome shift by mimicking OPN-KO with OPN neutralization via an antibody against OPN (OPNAb) and comparing the results to those obtained by using IgG as a control. When the HFD-fed mice were treated with OPN neutralization, we observed that the composition of the microbiota at the phylum level in the HFD-OPNAb group was similar to that in the HFD-KO group, which included the increased abundance of *Bacteroides* and *Proteobacteria*, compared to those in the HFD-IgG and HFD-WT groups (Fig. 5H). In addition, there was still a higher F/B ratio in the HFD-WT and HFD-IgG groups than in the HFD-KO and HFD-OPNAb groups (Fig. 5I). The above results collectively demonstrate that the administration of external OPN *in vitro* or *in vivo* is able to directly regulate the composition of the intestinal microbiota from an OPN-KO-like profile to a WT-like profile.

### OPN inhibited the adhesion of *Lactobacillus* to intestinal epithelial cells via Notch1 signaling.

**FIG 6:**
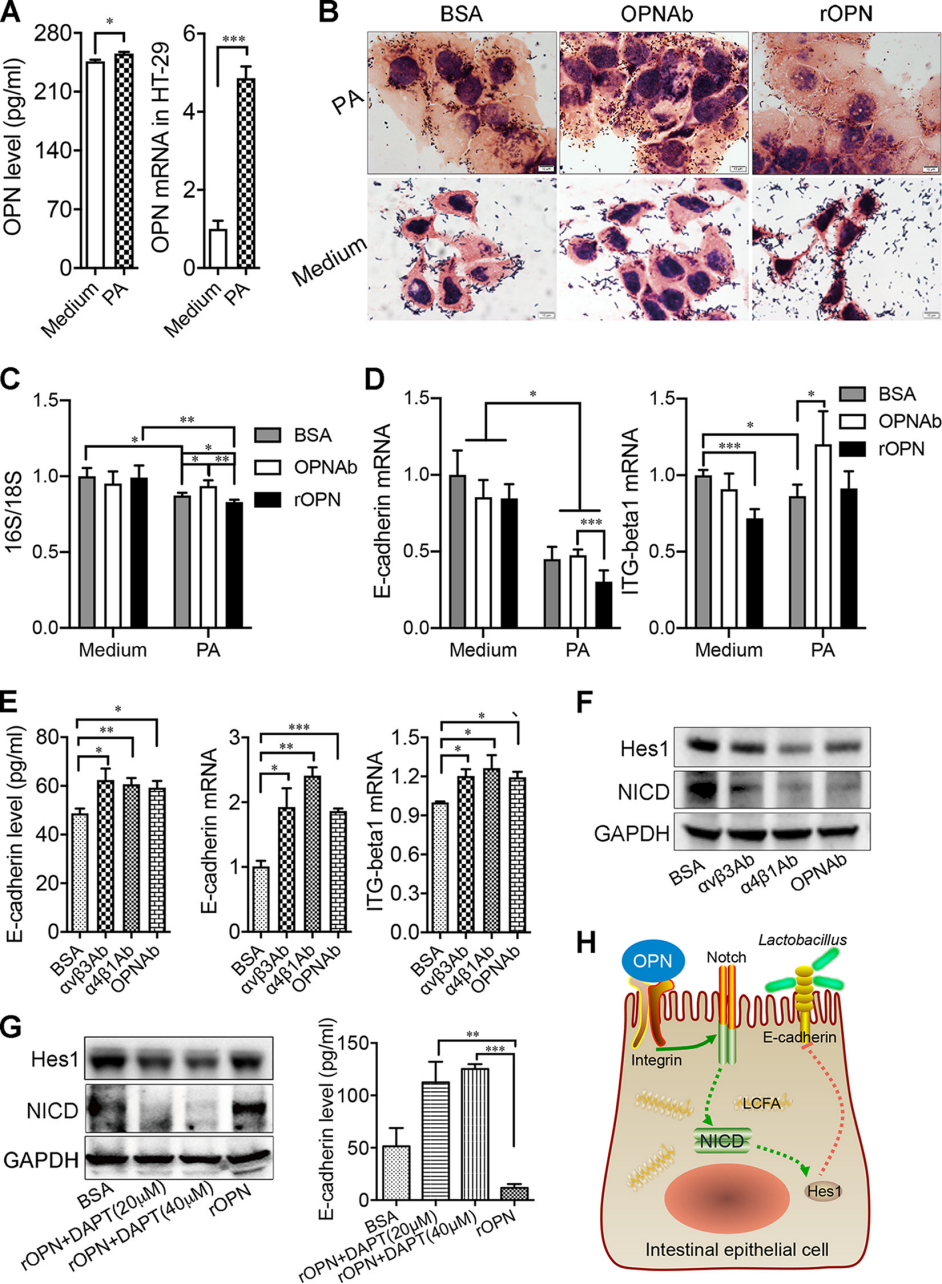
OPN decreased the adhesion of probiotic bacteria to intestinal epithelial cells treated with high fat. (A) The relative expression of OPN in HT-29 cells that were cultured with or without 200 μmol/L of palmitic acid (PA). (B) The representative images of *Lactobacillus* cultured with the OPN neutralizing antibody or with recombinant OPN in the medium containing PA or not. Scale bars, 50 μm. (C) The ratio of 16S and 18S, as well as (D) the relative expression of E-cadherin and integrin β were detected in the medium containing PA or not. When blocking integrin α4, the αv chain, and OPN, (E) the levels of E-cadherin and integrin β, as well as (F) the activation of Notch signaling were detected in the supernatant containing PA. (G) The activation of Notch1 or the expression of E-cadherin was measured after the administration of OPN with or without DAPT. Experiments were conducted in triplicate. (H) OPN facilitated the metabolism of fatty acids and HFD-induced metabolic disorders by regulating the composition of gut microbiota, including *Lactobacillus*, and their adhesion to the intestine via the adhesion molecules E-cadherin and the Notch signaling pathway. Data are given as the mean ± SEM. A Student’s *t* test (panel A) and a two-way ANOVA (panels C–E and G) were used to analyze the data with *n* = 4 in 3 experiments. *, *P* < 0.05; **, *P* < 0.01; ***, *P* < 0.001.

One of the major mechanisms of host factors modulating certain species of gut microbiota is via colonization regulation by affecting their adhesion to intestinal epithelial cells (21). When mice were treated with HFD, the mRNA of E-cadherin and integrin β were both higher in the OPN^−/−^ mice than in the WT mice. However, in the ND-WT and ND-KO groups, the mRNA of E-cadherin and integrin β showed no significant differences (Fig. S8A and B). To test whether this applies to the underlying mechanism for the contribution of OPN to the reduction of the dyslipidemia-resistant probiotics *Lactobacillus*, we investigated whether OPN could modulate the adhesion of *Lactobacillus* after the administration of palmitic acid (PA) *in vitro*. First, the expression of OPN was increased after the administration of PA (Fig. 6A). The adhesion of *Lactobacillus* was inhibited after the administration of PA. When the medium was supplemented with PA, the neutralization antibody of OPN facilitated the adhesion of *Lactobacillus* to intestinal epithelial cells, and this adhesion was mitigated by additional OPN (Fig. 6B and C). Furthermore, when exposed to PA, the levels of the adhesion molecules E-cadherin and integrin β were decreased compared to those of the controls. The levels of adhesion molecules E-cadherin and integrin β of the intestinal epithelial cells were significantly increased in the absence of OPN, while the administration of recombinant OPN (rOPN) inhibited the production of these two molecules (Fig. 6D). However, in the absence of PA, the adherence of *Lactobacillus* and the levels of the adhesion molecules remained unchanged (Fig. 6D). When blocking OPN or its receptor, integrins αv and α4, the levels of E-cadherin and integrin β were significantly increased (Fig. 6E). A previous study has shown that Notch signaling is involved in the production of adhesion molecules (22). To investigate the role of Notch signaling in OPN-regulated adhesion, we measured the activation of Notch signaling (cleaved Notch1, NICD) via Western blotting. The results showed that the expression levels of NICD and its target Hes1 were upregulated after the administration of rOPN, whereas this function of OPN was inhibited by the neutralization of integrins αv and α4 with the specific antibody or by the inhibition of NICD with DAPT (Fig. 6F and G). Indeed, the level of the adhesion molecule E-cadherin was increased in the presence of DAPT (Fig. 6G). Furthermore, we detected the levels of E-cadherin and integrin β via cytometry. When cultured with no PA, there was no difference in the mean fluorescence intensity (MFI) of E-cadherin (CD324) or integrin β (CD29) in the intestinal epithelial cells among these three groups. PA in the medium decreased the MFI of E-cadherin and integrin β. The MFI values of E-cadherin and integrin β were lower on the intestinal epithelium cells with the administration of rOPN than on those of the other two groups when cultured with PA. When intestinal epithelium cells cultured with PA were treated with αv or α4 antibodies or were in the presence of DAPT, the decrease of E-cadherin and integrin β MFI were inhibited (Fig. S8C and D). These results collectively suggested that OPN might induce the hyperlipidemia-mediated dysbiosis of gut microbiota by inhibiting the adhesion of the specific gut probiotic *Lactobacillus* to intestinal epithelial cells via the Notch signaling pathway in hyperlipidemia (Fig. 6H).

10.1128/mbio.02531-22.11 FIG S8 OPN decreased the adhesion of probiotic bacteria to intestinal epithelial cells treated with high fat. (A) The level of mRNA of E-cadherin and (B) integrin β was detected in WT and KO mice at week 24 with ND or HFD administration. (C–D) By flow cytometry, the MFI of E-cadherin and integrin β was detected in HT-29 cells cultured with OPN neutralizing antibody or recombinant OPN in medium containing PA or not. When blocking integrin α4, the αv chain, and OPN, cultured with or without DAPT, the levels of E-cadherin and integrin β were detected in the supernatant containing PA. Data given as mean ± SEM. A two-tailed Student’s *t* test (panels A, B, D) was used to analyze the data with *n* = 4 in 3 experiments. *, *P* < 0.05. Download FIG S8, TIF file, 0.6 MB. Copyright © 2022 Chen et al. 2022 Chen et al. https://creativecommons.org/licenses/by/4.0/ This content is distributed under the terms of the Creative Commons Attribution 4.0 International license.

## DISCUSSION

More attention has been paid recently to metabolic disorders and to the complications associated with gut microbiota (23–25). The intestinal microbiome is regulated by environmental, dietary, and host factors. OPN is known not only as an extracellular matrix protein but also as an immunoregulatory cytokine (8, 12, 26). Interestingly, we reported here, for the first time, that OPN regulated intestinal microbiota and thereby influenced HFD-induced metabolic disorders.

It was reported previously that metabolic disorders, including fatty liver disease and insulin resistance, were milder in OPN-deficient mice (13, 27). In our study, the OPN-deficient mice could resist HFD-induced metabolic disorders. However, OPN did not affect food intake. Instead, it influenced the process of lipid accumulation via the metabolism of LCFAs. SCFAs are also important in dietary-induced metabolic disorders (28–31). In our study, the level of SCFAs observed in the HFD-fed mice was significantly lower than that observed in the ND-fed mice. However, there was no significant difference between the HFD-WT mice and the HFD-KO mice, indicating that an OPN deficiency did not affect the metabolism of SCFAs.

We found that OPN could regulate intestinal microbiota, among which, the relative abundance of a main gas-producing bacterial species Dorea formicigenerans was positively correlated with the OPN levels as well as with the body weight. Our findings were in concordance with a recent study showing that *Dorea* was a positively BMI-related gut microbiota genus and was associated with LCFA accumulation in humans (2, 32). Raman et al. also reported a significant increase of *Dorea* in humans with nonalcoholic fatty liver disease (NAFLD) (33). The increased abundance of *Dorea* is in a possible association with the NAFLD-nonalcoholic steatosis heptitis (NASH) transition, and its clinical impact on patient outcomes has been demonstrated to be a higher prevalence of cirrhosis and hepatocellular carcinoma (HCC) in both people with obesity and patients with diabetes (34, 35). Furthermore, when the OPN-deficient offspring were foster-nursed by WT lactating mice, the microbiota of the OPN-deficient infants were similar to those of the WT infants, suggesting that feeding external OPN can directly change the composition of intestinal microbiota in OPN-deficient mice. Breast milk is a main and optimal food for newborn litters during the first 3 weeks after they are born and is an important source of fat and probiotics. This excludes the interplay of coprophagy to some extent. The gut microbiota is being originally established and matured in infants during these 3 weeks, and it is more easily influenced by food and by the environment than are those of adults. It was worth noting that the percentage of fat in mouse milk can be up to approximately 21.4% (36), which is close to that of the high-fat diet (31.5%) and significantly higher than that of the normal diet (about 4%). In fact, high fat in breast milk is required for an OPN-mediated change in the microbiome. On the other hand, this result also suggests a new mechanism by which breast milk affects the gut microbiome as a host factor.

Probiotics such as *Lactobacillus* were reported to improve metabolic disorders in a HFD mouse model, which was involved in epithelial barrier function and adherence junction protein expression, including the Notch signaling pathway and E-cadherins (37–39). Fig. 3 indicated that *Lactobacillus* could be the target bacterial genus in OPN-mediated lipid metabolism. In the top 25 genera, there was a statistically significant difference in the relative abundance of *Lactobacillus* and *Dorea* between the groups of ND-WT and HFD-WT as well as between the groups of HFD-WT and HFD-KO. The abundance of *Lactobacillus* is relatively higher than that of *Dorea* in the top 25 genera. In addition, previous studies showed that OPN could decrease the Gram-positive, including *Lactobacillus*, bacterial adhesion to control biofilm formation, which probably selectively decreased the adhesion of *Lactobacillus* to the intestinal epithelium (40). Furthermore, the administration of *Lactobacillus* induced a significant decrease in the relative abundance of pathogenic taxa, including *Dorea*, in rats (41, 42). In concordance with this report, we also found a negative correlation between the relative abundances of *Lactobacillus* and *Dorea* as well as that the abundance of *Dorea* was significantly decreased after the intragastric administration of *Lactobacillus* in HFD-fed mice. OPN may further regulate the abundance of *Dorea* and other dyslipidemia-promoting gut bacteria by reducing the abundance of the dyslipidemia-resistant, probiotic bacteria *Lactobacillus* in HFD-fed mice.

OPN interacts with a variety of cell surface receptors, including the αvβ3, αvβ5, and α4β1 integrins, as well as CD44 (12, 43, 44). The binding of OPN to these cell surface receptors stimulates cell adhesion and specific signaling functions. Importantly, OPN expression is negatively associated with E-cadherin expression. When blocking OPN or its receptor, integrins αv and α4, the levels of E-cadherin were significantly increased. In *in vitro* experiments, the lipid component with only PA in the medium is relatively pure, which is difficult to fully mimic in an *in vivo* high-fat diet. Indeed, there could be other mechanisms that interact with the adhesion results. In previous studies, MMP-2 has been implicated with E-cadherin in part proteolysis and cleavage (45); Also, E-cadherin localization to the cell-cell membrane was decreased when treated with TGF-β1 (46). HFD-induced TGF-β/Gbb signaling provokes insulin resistance by increasing tribbles expression and elevating MMP-2 levels in the skeletal muscles of C57BL/6J mice (47, 48). Bi et al. reported that OPN treatment could regulate Notch1 gene expression in cell fate determination and differentiation signals (49). However, our results showed that the Notch signaling pathway was upregulated after the administration of rOPN, whereas this function of OPN was inhibited by the neutralization of integrins αv and α4 with the specific antibody. Notch signaling activation was reported to inhibit the adipogenic differentiation of mesenchymal and adipose-derived stem cells that accelerated obesity (50). In this regard, it was shown here that OPN dampens the adhesion of the probiotic bacteria *Lactobacillus* to intestinal epithelial cells by inhibiting the production of adhesion molecules through the Notch signaling pathway.

In conclusion, our findings demonstrated for the first time that OPN could aggravate HFD-induced metabolic disorders. This can be due to the OPN-regulated shift of intestinal microbiota, which was further verified in the experiments of *in vivo* cross-fostering and *in vitro* fecal bacterial culture. OPN also regulated the adhesion of the probiotic bacteria *Lactobacillus* to intestinal epithelial cells via adhesion molecules in a high-fat microenvironment (Fig. S9). OPN may further upregulate the abundance of the gas-producing bacteria *Dorea* by reducing the intestinal adhesion of the probiotic bacteria *Lactobacillus*, leading to HFD-induced dyslipidemia. At present, there are few reports indicating that a single host protein can directly affect the gut microbiome, which provides a novel method for the regulation of the gut microbiome. Therefore, OPN, as well as its regulated *Dorea* and *Lactobacillus* species, may serve as a potential pharmaceutical target for weight control in terms of the prevention and treatment of metabolic disorders. The application prospects of OPN in regulating the gut microbiome remain profound in the future.

10.1128/mbio.02531-22.12 FIG S9 Graphic abstract. OPN facilitated the metabolism of fatty acids and HFD-induced metabolic disorders by regulating the composition of gut microbiota and their adhesion to the intestinal epithelium via the adhesion molecules E-cadherin and Notch signaling. Download FIG S9, TIF file, 1.4 MB. Copyright © 2022 Chen et al. 2022 Chen et al. https://creativecommons.org/licenses/by/4.0/ This content is distributed under the terms of the Creative Commons Attribution 4.0 International license.

## MATERIALS AND METHODS

### Participants.

A total of 28 individuals with obesity were enrolled in the First Affiliated Hospital, School of Medicine, Zhejiang University, China, under the ethical approval of the Clinical Research Ethics Committee of the First Affiliated Hospital, College of Medicine, Zhejiang University, China (ref. 2020-110). Consent was obtained from the individuals prior to the procedure after a full explanation of the purpose of the study. Obesity was defined here as a body mass index (BMI) of 25 kg/m^2^ or greater in the study. The details for all of the participants are provided in Table 2.

**TABLE 2:** Demographic and clinical characteristics of the participants in study cohorts^*a*^

Groups	N	Age (Median, y)	Height (median, cm)	Weight (median, Kg)	Body mass index (mean)
Obesity					
Male	20	47 (24 to 75)	170.5 (160 to 79)	77.6 ± 7.65	26.71 ± 6.49
Female	8	60 (31 to 76)	157 (150 to 171.5)	67.9 ± 10.04	27.08 ± 7.08
Healthy control					
Male	18	55 (32 to 70)	166 (153.7 to 175.5)	62.5 ± 4.98	22.70 ± 2.03
Female	9	57 (29 to 68)	162.5 (150.5 to 170)	53.5 ± 3.32	20.21 ± 1.39

a Patients diagnosed with cancer, major infectious diseases, cardiovascular diseases, or autoimmune diseases were excluded.

### Animals.

All of the procedures were performed according to the “Guide for the Care and Use of Laboratory Animals” published by the National Institutes of Health (publication 86–23 revised 1985) and under the ethical approval of the Committee of Animal Experimental Ethical Inspection of the First Affiliated Hospital, College of Medicine, Zhejiang University, China. The specific pathogen-free female mice and OPN-deficient mice (B6.129S6(Cg)-*Spp1^tm1Blh^*/J) were both of the same background (C57BL/6J) and were both originally from Jackson Laboratory (Bar Harbor, ME, USA). 4-week-old female wild-type (WT) mice and OPN-deficient (KO) mice were sacrificed after a continuous, 24-week high-fat diet (HFD, 60% kcal of fat, cat. number: D12492; Research Diets, Inc.) or a normal diet (ND) containing approximately 4% kcal of fat.

### Measurement of serum parameters.

Glucose concentration was determined using the ACCU-CHEK (ROCHE, Switzerland) glucose analyzer. The levels of triglycerides (TG), total cholesterol (TC), high density lipoprotein (HDL), and low density lipoprotein (LDL) were determined using a DRI-CHEM 4000ie (Fujifilm, Japan). An enzyme-linked immunosorbent assay (ELISA) was used for measuring the levels of blood insulin (ALPCO, USA), OPN, and leptin (R&D Systems, USA).

### Metagenomic sequencing analysis.

Total genomic DNA was extracted from fecal samples using a modified CTAB method, as described previously (Novogene, China) (51). All of the samples were sequenced based on the Novaseq PE150 platform (Illumina, USA). 12 G raw paired-end sequencing reads were processed using the MOCAT software package. Reads were assembled into scaftigs and mapped to the reference gene catalog using the SOAP aligner v2.21 package in MOCAT (52). Catalog genes were assigned taxonomical annotations based on their sequence similarity to a database of predicted protein coding genes from the National Center for Biotechnology Information (NCBI, release 196) by MyTaxa (53). The taxonomic abundance was calculated based on the gene abundance. The results were presented for annotations against the KEGG (Kyoto Encyclopedia of Genes and Genomes) ortholog (KO) hierarchy.

### 16S rDNA sequencing analysis.

Total genomic DNA from samples was extracted for amplification using the V3 to V4 regions (51). Sequencing libraries were prepared using a TruSeq DNA PCR-Free Sample Preparation Kit (Illumina, USA), following the manufacturer's recommendations. The library quality was evaluated on a Qubit 2.0 Fluorometer (Thermo Scientific, USA) and an Agilent Bioanalyzer 2100 system (Agilent Technologies, USA). At last, the library was sequenced on an Illumina HiSeq2500 platform (Illumina, USA), and 250 bp paired-end reads were generated. The 16S rDNA gene sequence data were processed using QIIME (V1.7.0) with the default parameters (54). Sequence analyses was performed using the Uparse software package (Uparse v7.0.1001) (55). Sequences with ≥97% similarity were assigned to the same operational taxonomic units (OTUs). The representative sequence for each OTU was screened for further annotation. The output data were further analyzed using the Statistical Analysis of Metagenomic Profiles (STAMP) software package (version 2.1.3).

The correlation between the different bacterial genera was calculated using the Pearson correlation. This correlation coefficient quantifies the strength of correlation (from −1 to 1), and a coefficient with absolute value of greater than 0.5 is regarded as a strong correlation. The networks were drawn using the Cytoscape software package (v3.7.0).

### Fecal bacterial isolates and culture *in vitro*.

The ileocecal contents of two normal WT mice were collected, rinsed with sterile PBS, and centrifuged at 200 g for 5 min. The supernatant was collected, and an anaerobic culture of the supernatant was performed at 37°C in hemolysin-containing anaerobic bottles (labeled as the Hab group). Recombinant OPN protein (R&D Systems, USA) was added to each culture medium for 24 h (labeled as the Hab-rOPN group), and bovine serum albumin (BSA) was added as a control.

### Cross-fostering.

Newborn litters from WT and OPN-deficient mice were split and swapped, respectively, within 24 h of birth (labeled as KO-WT and WT-KO). Accordingly, controls without swapping were labeled as KO-KO and WT-WT. The mice from all four of the groups were weaned after 3 weeks, and this was followed by an analysis of their gut microbiota.

### Fecal microbiota transplantation.

FMT was performed in the mice as follows. The WT mice received a 2 week treatment of antibiotics (1 g/L of ampicillin, 1 g/L of neomycin, 1 g/L of metronidazole, and 0.5 g/L of vancomycin) in drinking water prior to the FMT. Pseudosterile mice were transplanted with 20 mg of fresh feces collected after 20 to 24 weeks on the HFD and resuspended in 200 μL sterile PBS from the same and cohousing HFD-KO donor or HFD-WT donor once daily for a week. Then, all of the mice were fed with the HFD for 3 months. To reinforce the microbiota genotype, fecal microbiota were given weekly throughout the study.

### Bacterial adhesion assay in intestinal epithelial cells.

For of bacterial adhesion experiments, HT-29 cells (ATCC number: HTB-38) were cultured in McCoy’s medium with palmitic acid (200 μM, Sigma-Aldrich, USA) for 14 h at 37°C with 5% CO_2_. *Lactobacillus* was cultured aerobically in brain heart infusion (BHI) broth (MRS, Oxoid, Basingstoke, UK) at 37°C. The bacteria were harvested, washed, and resuspended in sterile phosphate-buffered saline (PBS) and the colony forming units (CFU) were measured on BHI agar plates that were incubated at 37°C.

Then, the HT-29 cells were cocultured with 5 × 10^7^ CFU/mL bacteria for 2 h with or without a 2 h preintervention of an anti-OPN antibody (R&D Systems, USA, 1 μg/mL) and the recombinant OPN (rOPN) protein (R&D Systems, USA, 1 μg/mL). Subsequently, Gram-stained cross-sections of embedded and fixed cells with adhering bacteria were microscopically examined to allow for the enumeration of the number of adhering bacteria for the two membrane regions distinguished. Host and bacterial DNA were extracted for quantitative PCR analysis. OPN or its receptor integrins αvβ3 (R&D Systems, USA) and α4 (R&D Systems, USA) were blocked with neutralizing antibodies. The rOPN, OPNAb, and αv, α4 antibodies were dissolved in PBS containing 0.5% BSA. The activation of Notch1 was measured in the presence of rOPN or a γ-secretase inhibitor (DAPT, Beyotime, China) via Western blotting. The levels of adhesion molecules E-cadherin and integrin β were measured via qPCR or ELISA in the presence of DAPT.

### Statistical analysis.

The GraphPad Prism 6.01 software package was used for the statistical analyses. All data are given as means ± standard errors of the mean (SEMs). Two-way analyses of variance (ANOVA), Student’s *t* tests (and nonparametric tests), or Mann-Whitney tests were performed to determine statistical significance. For the correlation analyses, Spearman’s rank correlation test was used. A P value of <0.05 is regarded as indicative of a statistically significant result.

### Data availability.

All authors have access to the study data and have reviewed and approved the final manuscript. The original data set was deposited in the NCBI GEO database (https://www.ncbi.nlm.nih.gov/geo/) under the accession numbers GSE216386. Additional methods can be found in Text S1 in the supplemental material.

10.1128/mbio.02531-22.4 TEXT S1 Supplementary materials and methods. Download Text S1, DOCX file, 0.03 MB. Copyright © 2022 Chen et al. 2022 Chen et al. https://creativecommons.org/licenses/by/4.0/ This content is distributed under the terms of the Creative Commons Attribution 4.0 International license.
