# Supplementary material for: Osteopontin Exacerbates High-Fat Diet-Induced Metabolic Disorders in a Microbiome-Dependent Manner
Source: mBio. 2022 Oct 27;13(6):e02531-22. doi: 10.1128/mbio.02531-22 (PMC9765578; doi:10.1128/mbio.02531-22)
Supplement: TEXT S1 [file mbio.02531-22-s0001.docx]

**Supplementary Information for**

Osteopontin Exacerbates High-Fat Diet-induced Metabolic Disorders in a microbiome-dependent Manner

**Supplementary methods and materials**

**Analysis of fat distribution**

Fat distribution was measured by T1-weighted magnetic resonance imaging (MRI) with QMR23-060H-I imaging instrument (Suzhou Niumag Analytical Instrument Corporation, China) following the manufacturer’s instruction. Mice were anesthetized with 10% chloral hydrate (4 mL/kg) and then placed in a 40 mm probe coil to detect the coronal image. The fat highlight image was analyzed by ImageSystem (Suzhou Niumag Analytical Instrument Corporation, China).

**Intestinal transcriptome analysis**

Total RNA was extracted from the intestine tissues using TRIzol (Takara, Japan). RNA was then qualified, enriched and randomly interrupted by divalent cations in NEB Fragmentation Buffer (New England Biolabs, USA). Upon completion of library construction, the Qubit2.0 Fluorometer and Agilent 2100 bioanalyzer system were applied to evaluate the library, followed by qRT-PCR to accurately quantify the effective concentration of the library. The sequencing fragment was transformed from image data to sequence data. HISAT2 software was used to conduct genomic localization analysis on the filtered sequences, and after the alignment of clean reads to the genome or transcriptome, relevant calculations of reads count could be made to understand the gene expression. According to the expression level (fragments per kilobase of transcript per million mapped reads, FPKM) of all genes in each sample, the correlation coefficient was compared to investigate the inter-group difference. Principal component analysis (PCA) was used to evaluate the difference between groups. DEseq2 software was used to analyze the expression of differentially expressed genes, and the volcano map, Venn diagram and cluster map of differentially expressed genes were drawn. The clusterProfiler software package was used to carry out GO function enrichment analysis and KEGG pathway enrichment analysis on the differential gene, and to find out which biological functions or pathways the differential genes under different conditions were significantly correlated with.

**Glucose tolerance test, insulin tolerance test and oral lipid tolerance test**

Glucose tolerance test (GTT) was performed in mice with an overnight fasting period. Blood glucose was measured before and after an intraperitoneal injection of glucose (1 g/kg body weight, Sigma-Aldrich, USA) at the indicated time point. For insulin tolerance test (ITT), mice were fasted overnight. Following an intraperitoneal bolus injection of recombinant human regular insulin (1 U/kg body weight, Novolin R, Novo Nordisk Inc., USA), blood glucose concentrations were measured before and at 30, 60, 90, and 120 minutes after injection. Oral lipid tolerance test (OLTT) was conducted after a fasting of 24 h. During OLTT, the ingestion of pure water was allowed over the 6 hours. Then, mice were treated with 1h of high fat diet. Blood samples were drawn at 0h (fasting), 1h, 2h, 4h, and 6 h.

**Measurement of long-chain fatty acids**

Long-chain fatty acids (LCFAs) were measured as follow ([1](#_ENREF_1)). A 50 mg sample was dissolved in the internal standard solution (C17:0 fatty acid methyl ester) (Shanghai Metabolome Institute-Wuhan, China), adding methyl ester reagent reaction, n-hexane solvent extracting and then drying. The sample was further dissolved in 50 μL n-hexane for gas chromatography mass spectrometry (GC-MS) analysis. GC-MS analysis was performed with GC-MS 2010 plus chromatographic system equipped with a hydrogen ion flame detector (Shimadzu Corporation, Japan). The total fatty acids were measured quantitatively and qualitatively by the internal standard method using GC Solution software (Shimadzu Corporation, Japan).

**Flow cytometry**

FITC-anti-human CD324 (67A4, Biolegend, USA) and PE-anti-human CD29 (TS2/16, Biolegend, USA) were applied for flow cytometry. Stained cells were assessed on a FACS Canto II instrument (BD Immunocytometry Systems, USA) for data acquisition. Data were analyzed with FlowJo (BD Immunocytometry Systems, USA).

**Statistical analysis**

GraphPad Prism 6.01 software was used for statistical analyses. All data are expressed as means ± SEM. Two-way ANOVA, Student *t* test (and nonparametric tests) or Mann-Whitney test were performed to determine significance. For correlation analyses, Spearman's rank correlation test was used. A stastistically significance of differences was considered when *P* < 0.05.

**References**

1. An Y, Xu W, Li H, Lei H, Zhang L, Hao F, Duan Y, Yan X, Zhao Y, Wu J, Wang Y, Tang H. 2013. High-fat diet induces dynamic metabolic alterations in multiple biological matrices of rats. J Proteome Res 12:3755-68.
